# MicroRNA-34a expression levels in serum and intratumoral tissue can predict bone metastasis in patients with hepatocellular carcinoma

**DOI:** 10.18632/oncotarget.13531

**Published:** 2016-11-23

**Authors:** Zuo-Lin Xiang, Xiao-Mei Zhao, Li Zhang, Ping Yang, Jia Fan, Zhao-You Tang, Zhao-Chong Zeng

**Affiliations:** ^1^ Department of Radiation Oncology, Zhongshan Hospital, Fudan University, Shanghai, China; ^2^ Department of Liver Cancer Institute, Zhongshan Hospital, Fudan University, Shanghai, China

**Keywords:** hepatocellular carcinoma, bone metastasis, microRNA-34a, serum, tissue microarray

## Abstract

Hepatocellular carcinoma (HCC) patients with bone metastasis (BM) suffer from pain and other symptoms that significantly reduce their quality of life. We screened a microRNA (miRNA) microarray to identify potential serum biomarkers for BM in HCC patients. A miRNA microarray was used to screen for BM-related miRNAs in paired serum samples from HCC patients with BM and from HCC patients without BM. Real-time quantitative polymerase chain reaction (qRT-PCR) was used to quantify candidate miRNAs in serum samples from 106 independent HCC patients. Levels of candidate miRNAs in tissue samples from an independent cohort of 296 HCC patients were evaluated by *in situ* hybridization and intratumoral tissue microarray. The migration and invasion capabilities of HCCLM3 and SMMC-7721 cells were evaluated following treatment with a mimic and an inhibitor of miR-34a. Ninety miRNAs were differentially expressed in sera from HCC patients with BM when compared with sera from non-BM HCC patients (*P* < 0.05). Only miR-34a and miR-498 had false discovery rates (FDRs) < 0.05. In cohorts of 106 and 296 HCC patients, we found that reduced serum and intratumoral miR-34a expression levels were independent risk factors for developing BM. Migration and invasion experiments indicated that a reverse correlation existed between miR-34a and HCC tumor migration and invasion. This study demonstrates the potential for the use of miR-34a as a serum and intratumoral tissue biomarker for predicting the risk of BM in HCC patients.

## INTRODUCTION

Hepatocellular carcinoma (HCC) has an extremely poor prognosis and is the fifth most common cancer in men and the ninth in women worldwide. It is also the second leading cause of cancer death in men and the sixth in women worldwide [1]. More than 80% of HCC cases occur in sub-Saharan Africa and in Eastern Asia [2], and most are associated with liver cirrhosis as a result of chronic hepatitis B virus (HBV) or hepatitis C virus (HCV) infection. The frequency of bone metastasis (BM) in HCC patients with extrahepatic metastases was estimated to be 38.5% [3], whereas the frequency of BM in HCC patients that underwent curative resections was 11.7% [4]. During early metastasis, bone is destroyed by osteoclasts, which can cause pathological fractures, severe pain, and other nerve compression syndromes [5]. Bisphosphonates can inhibit the activity of osteoclasts and are used to reduce the frequency and severity of skeletal complications in BM patients [6]. Early prediction or diagnosis of BM in HCC patients is important for designing appropriate therapeutic regimens and assessing prognoses. If we can identify biomarkers to predict BM in HCC patients, we could take measures to reduce the probability that BM will develop and potentially enhance the quality of life for HCC patients.

We have determined that intratumoral connective tissue growth factor (CTGF), interleukin-11 (IL-11), and CXCR4 overexpression in primary tumor tissues is associated with BM in HCC patients [4, 7, 8]. We have also established a clinicopathologic model to predict BM in HCC patients [9], but this model is based on intratumoral tissue, which is inaccessible in many HCC patients. On the other hand, a serum biomarker screen combined with a predictive model could be used for every HCC patient. Further, microRNA (miRNA) is more stable than mRNA in formalin-fixed, paraffin-embedded (FFPE) specimens and is easily obtained from patient serum. Thus, we investigated whether serum and intratumoral tissue miRNAs could be used to predict BM in HCC patients.

## RESULTS

### Serum miRNA microarray analysis

**Figure 1:**
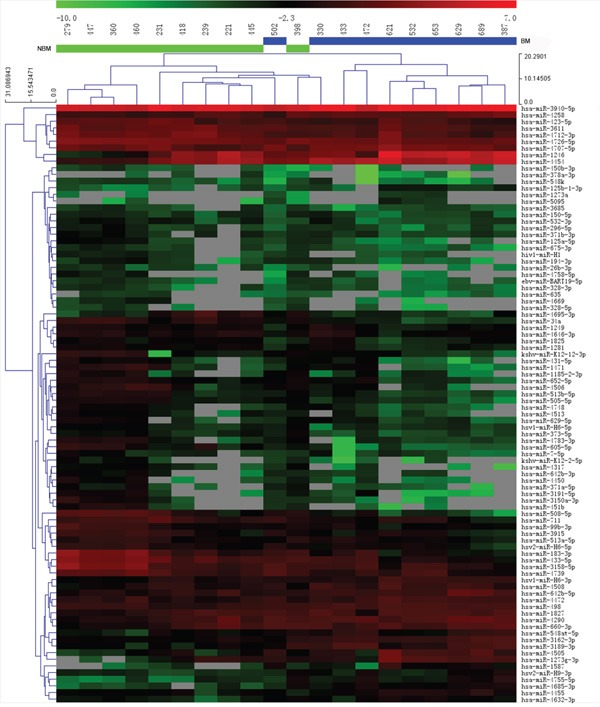
Hierarchical clustering of 90 differentially expressed miRNAs Hierarchical clustering of miRNAs that were differentially expressed in the sera of HCC patients with BM vs. non-BM HCC patients. Red indicates high relative expression and green indicates low relative expression.

We identified 90 miRNAs that were differentially expressed in the BM and non-BM groups (Supplementary Table S1). In serum samples from the BM group, 27 miRNAs were upregulated and 63 miRNAs were downregulated when compared with serum samples from the non-BM group (Figure 1). Among the 90 miRNAs, only two had FDR values<0.05; miR-34a was downregulated and had an FDR of 0.020 and miR-498 was upregulated and had an FDR of 0.018. MiR-34a and miR-498 were further analyzed in an independent cohort of 106 HCC patients to determine whether they could serve as potential serum biomarkers to predict BM.

### qPCR confirmed serum miR-34a as a risk factor for BM

To further evaluate the potential of miR-34a and miR-498 to serve as biomarkers, qPCR was performed to quantify their levels in the sera of 106 HCC patients. The results of the univariate analyses revealed that serum miR-34a (*P*=0.002), serum miR-498 (*P*=0.038), tumor differentiation (*P*=0.011), tumor number (*P*=0.035), vascular invasion (*P*=0.005), and BCLC stage (*P*=0.002) were risk factors for BM in HCC patients (Table 1). The results of the multivariate analyses revealed that serum miR-34a (*P*=0.010), vascular invasion (*P*=0.047), and BCLC stage (*P*=0.039) were independent risk factors for BM in HCC patients (Table 2). Of the two miRNAs, only miR-34a predicted BM in HCC patients. At follow-up, 14 of the 106 HCC patients developed BM. Among the 14 BM patients, 13 patients exhibited down-regulated miR-34a levels.

**Table 1:** Univariate analyses of factors associated with bone metastasis in a cohort of 106 HCC patients

Variable	Bone metastasis	
	HR (95% CI)	*P*
Age (≤ 51 versus >51 years)	2.488 (0.779-7.943)	0.124
Gender (male versus female)	0.834 (0.186-3.739)	0.813
HBsAg (negative versus positive)	3.824 (0.499-29.277)	0.197
HCV-Ab (negative versus positive)	3.636 (0.467-28.289)	0.218
AFP, ng/mL (≤ 20 versus >20)	0.628 (0.210-1.876)	0.404
ALT, U/L (≤ 40 versus >40)	1.188 (0.412-3.427)	0.750
γ-GT, U/L (≤ 50 versus >50)	1.549 (0.486-4.942)	0.460
Liver cirrhosis (no versus yes)	2.631 (0.343-20.171)	0.352
Child-Pugh score (A versus B)	0.048 (0.000-7.243E6)	0.752
Tumor differentiation (I–II versus III–IV)	3.954 (1.323-11.810)	0.011
Tumor size, cm (≤ 5 versus >5)	2.186 (0.730-6.545)	0.162
Tumor number (single versus multiple)	3.120 (1.081-8.999)	0.035
Tumor encapsulation (none versus complete)	1.227 (0.430-3.502)	0.702
Vascular invasion (no versus yes)	4.611 (1.599-13.296)	0.005
BCLC stage (0-A versus B-C)	5.599 (1.916-16.363)	0.002
Serum miR-34a (high versus low)	26.722 (3.478-205.288)	0.002
Serum miR-498 (low versus high)	3.870 (1.078-13.887)	0.038

NOTE: Univariate analysis and Cox proportional hazards regression model. Tumor differentiation evaluated was based on the Edmondson–Steiner grading.

Abbreviation: HBsAg, hepatitis B virus surface antigen; HCV-Ab, hepatitis C virus antibody; AFP, alpha-fetoprotein; ALT, alanine aminotransferase; γ-GT, γ-glutamyl-transferase; BCLC, Barcelona Clinic Liver Cancer

**Table 2:** Multivariate analyses of factors associated with bone metastasis in a 106 hepatocellular carcinoma patient cohort

Variable	Bone metastasis	
	HR (95% CI)	*P*
Tumor differentiation (I–II versus III–IV)	1.751 (0.552-5.556)	0.342
Tumor number (single versus multiple)	1.728 (0.540-5.528)	0.356
Vascular invasion (no versus yes)	3.078 (1.013-9.351)	0.047
BCLC stage (0-A versus B-C)	3.471 (1.068-11.286)	0.039
Serum miR-34a (high versus low)	16.034(1.915-134.265)	0.010
Serum miR-498 (low versus high)	1.978 (0.442-8.839)	0.372

NOTE: Variables were adopted for their prognostic significance on univariate analysis. Tumor differentiation evaluated was based on the Edmondson–Steiner grading.

Abbreviation: BCLC, Barcelona Clinic Liver Cancer.

### Correlations between miR-34a expression in intratumoral tissue and clinicopathologic features

ISH and intratumoral TMAs from 296 HCC patients were used to determine whether intratumoral miR-34a could predict BM in HCC patients. MiR-34a was primarily localized to the cytoplasm and nuclei of tumor cells (Supplementary Figure S1). Intratumoral miR-34a was expressed in 219 (74.0%) patients. The associations between miR-34a expression and clinicopathologic factors were analyzed (Supplementary Table S2). At follow-up, 91 patients developed extrahepatic metastases. Of these 91 patients, 37 developed BM, 46 developed lung metastasis, 39 developed lymph node metastasis, 9 developed adrenal gland metastasis, and 3 developed brain metastasis. Patients with multiple organ metastases were included in every applicable organ group. We analyzed whether or not there was a correlation between the expression of miR-34a and different organ metastasis and determined that intratumoral miR-34a expression was negatively correlated with BM (*r*= - 0.405; *P*< 0.001), but was not correlated with lung metastasis (*r*= - 0.094; *P*=0.105), lymph node metastasis (*r*= - 0.087; *P*=0.137), adrenal gland metastasis (*r*= -0.130; *P*=0.824), or brain metastasis (*r*= - 0.380; *P*=0.512). Intratumoral miR-34a expression was also correlated with BCLC stage (*r* = - 0.115; *P*= 0.048) and vascular invasion (*r*= - 0.121; *P*= 0.038), but did not correlate with other clinicopathologic factors (age, gender, HBsAg, HCV-Ab, AFP, ALT, γ-GT, liver cirrhosis, Child-Pugh score, tumor differentiation, tumor size, tumor number, or tumor encapsulation). We also found that BCLC stage (*r*= 0.194; *P*= 0.001), vascular invasion (*r*= 0.256; *P*< 0.001), tumor number (*r*= 0.118; *P*= 0.042), and tumor differentiation (*r*= 0.163; *P*<0.005) were positively correlated with BM.

### Correlations between intratumoral miR-34a expression and BM, non-BM, and overall metastasis

At the last follow-up for the group of 296 HCC patients, 91 (30.7%) of the patients presented with extrahepatic metastases including 37 (12.5%) with BM. Among the 37 BM patients, 31 patients exhibited down-regulated miR-34a levels. At five years, the BM-free survival rate was 49.9%, the progression-free survival rate was 45.9%, and the overall survival rate was 50.5%. The results of the log-rank test indicated that a lower intratumoral miR-34a expression level was more highly associated with increased incidence of BM than a higher miR-34a expression level (*P*<0.001). The median BM free survival time determined by the Kaplan-Meier and log-rank tests in patient groups with high miR-34a expression was 39.9 months, whereas the corresponding survival time in patients with low miR-34a expression was 17.8 months (*P*<0.001).

Cox regression univariate analyses revealed that tumor differentiation (*P*=0.011), tumor number (*P*=0.032), vascular invasion (*P*<0.001), BCLC stage (*P*=0.001), and the intratumoral miR-34a level (*P*<0.001) were associated with BM (Table 3). Cox regression multivariate analyses identified vascular invasion (*P*=0.003), BCLC stage (*P*=0.011), and the intratumoral miR-34a level (*P*<0.001) as independent risk factors for BM (Table 4).

**Table 3:** Univariate analyses of factors associated with bone metastasis in a cohort of 296 HCC patients

Variable	Bone metastasis	
	HR (95% CI)	*P*
Age (≤ 51 versus >51 years)	0.867 (0.454-1.655)	0.665
Gender (male versus female)	0.808 (0.286-2.280)	0.687
HBsAg (negative versus positive)	1.244 (0.547-2.834)	0.602
HCV-Ab (negative versus positive)	3.455 (0.333-18.094)	0.378
AFP, ng/mL (≤ 20 versus >20)	1.061 (0.524-2.148)	0.869
ALT, U/L (≤ 40 versus >40)	0.885 (0.455-1.720)	0.719
γ-GT, U/L (≤ 50 versus >50)	1.518 (0.760-3.034)	0.237
Liver cirrhosis (no versus yes)	2.138 (0.656-6.965)	0.207
Child-Pugh score (A versus B)	0.049 (0.000-5.708E5)	0.717
Tumor differentiation (I–II versus III–IV)	2.304 (1.209-4.391)	0.011
Tumor size, cm (≤ 5 versus >5)	1.546 (0.806-2.965)	0.190
Tumor number (single versus multiple)	2.041 (1.064-3.915)	0.032
Tumor encapsulation (none versus complete)	0.690 (0.358-1.331)	0.268
Vascular invasion (no versus yes)	4.516 (2.362-8.632)	<0.001
BCLC stage (0-A versus B-C)	3.198 (1.627-6.285)	0.001
Intratumoral miR-34a (positive versus negative)	8.005 (3.874-16.541)	<0.001

NOTE: Univariate analysis and Cox proportional hazards regression model. Tumor differentiation evaluated was based on the Edmondson–Steiner grading.

Abbreviation: HBsAg, hepatitis B virus surface antigen; HCV-Ab, hepatitis C virus antibody; AFP, alpha-fetoprotein; ALT, alanine aminotransferase; γ-GT, γ-glutamyl-transferase; BCLC, Barcelona Clinic Liver Cancer.

**Table 4:** Multivariate analyses of factors associated with bone metastasis in a 296 hepatocellular carcinoma patient cohort

Variable	Bone metastasis	
	HR (95% CI)	*P*
Tumor differentiation (I–II versus III–IV)	1.613 (0.822-3.163)	0.164
Tumor number (single versus multiple)	1.707 (0.848-3.434)	0.134
Vascular invasion (no versus yes)	2.773 (1.427-5.387)	0.003
BCLC stage (0-A versus B-C)	2.464 (1.226-4.949)	0.011
Intratumoral miR-34a(positive versus negative)	6.011 (2.857-12.645)	<0.001

NOTE: Variables were adopted for their prognostic significance on univariate analysis. Abbreviation: BCLC, Barcelona Clinic Liver Cancer.

For the 54 patients who developed metastases (lung, lymph node, adrenal gland, and brain metastases) that did not involve the bone (non-BM), Kaplan-Meier and log-rank tests indicated that the level of intratumoral miR-34a expression did not affect the rate of metastases in non-BM patients (*P*=0.521). The rate of overall metastases was higher in HCC patients with lower levels of miR-34a expression (*P*<0.001).

### MiR-34a negatively regulates HCC cell proliferation, migration, and invasion

Among the four HCC cell lines (SMMC-7721, HepG2, MHCC97H, and HCCLM3) with an ascending order of metastatic potentials (Supplementary Figure S2A), we chose SMMC-7721 cells, which exhibited the lowest degree of invasion and HCCLM3 cells, which exhibited the highest degree of invasion, for the migration and invasion assay. Further, their endogenous level of miR-34a was reversely attenuated (Supplementary Figure S2B), which indicated miR-34a may affect HCC metastasis.

MiR-34a expression in cells was induced or inhibited by transfection with a miR-34a mimic or a miR-34a inhibitor, respectively. As well, the results of the qPCR analysis demonstrated that transfection with 100 nM of the miR-34a inhibitor decreased miR-34a expression 5.0-fold in the SMMC-7721 cells (Supplementary Figure S2C), whereas transfection with 100 nM of the miR-34a mimic increased miR-34a expression 5.7-fold in the HCCLM3 cells (Supplementary Figure S2D).

**Figure 2:**
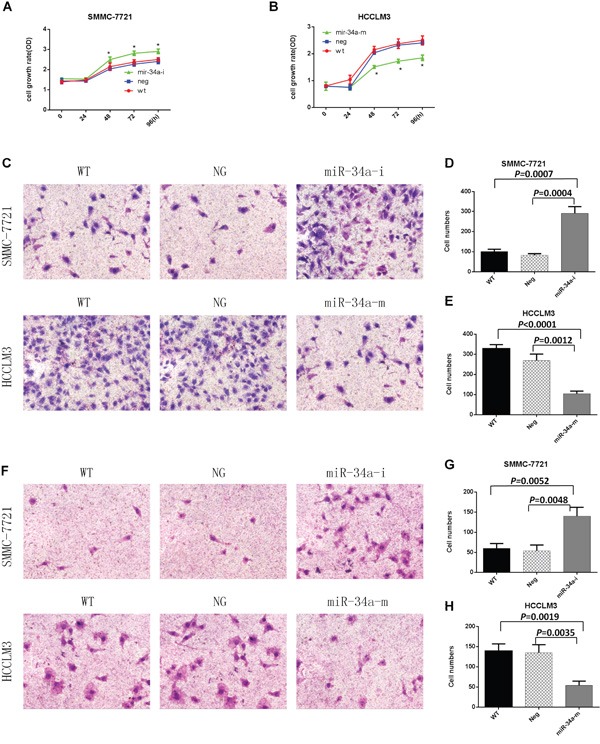
Proliferation, migration, and invasion assays of SMMC-7721 and HCCLM3 cells after transfection with the indicated oligonucleotides WT, NG, miR-34a-m, and miR-34a-i indicate non-transfected, negative control oligonucleotide-transfected, miR-34a mimic- transfected, and miR-34a inhibitor-transfected cells, respectively. **A**. SMMC-7721 cell growth rates after transfection with the miR-34a inhibitor. **B**. HCCLM3 cell growth rates after transfection with the miR-34a mimic. * = *P*<0.05, compared to negative controls at the same time point. **C**. Representative images of the migration assay. **D**. and **E**. Numbers of migrating cells on the undersides of the membranes. Data are presented as mean ± SEM and are representative of three independent experiments. The images were acquired at a magnification of 200×. **F**. Representative images of the invasion assay. **G**. and **H**. Numbers of invading cells on the undersides of the membranes. Data are presented as mean ± SEM and are representative of three independent experiments. The images were acquired at a magnification of 200×.

To study the role miR-34a played in HCC, we first investigated the effect of miR-34a on HCC proliferation using the Cell Counting Kit-8 assay, the results of which indicated that compared with the negative controls, the ability of cells to proliferate was slightly accelerated when the SMMC-7721 cells were transfected with the miR-34a inhibitor, whereas the proliferation ability was significantly inhibited when the HCCLM3 cells were transfected with the miR-34a mimic (Figure 2A, 2B). These findings indicated that miR-34a negatively regulated HCC cell proliferation.

In the SMMC7721 and HCCLM3 cell migration assays, the migration capabilities of the SMMC-7721 cells were increased 3.51-fold after transfection with the miR-34a inhibitor compared with negative control oligonucleotide-transfected SMMC-7721 cells (*P*=0.0004; Figure 2C, 2D). After transfection with the miR-34a mimic, the migration capabilities of the HCCLM3 cells were reduced 2.58-fold compared with the migration of the negative control oligonucleotide-transfected HCCLM3 cells (*P*=0.0012; Figure 2C, 2E).

In invasion assays with SMMC7721 and HCCLM3 cells, the invasion capabilities of the SMMC-7721 cells were increased 2.59-fold after transfection with the miR-34a inhibitor compared with negative control oligonucleotide-transfected SMMC-7721 cells (*P*=0.0048. Figure 2F, 2G). After transfection with the miR-34a mimic, the invasion capabilities of HCCLM3 cells were reduced 2.50-fold compared with the invasion of the negative control oligonucleotide-transfected HCCLM3 cells (*P* = 0.0035 Figure 2F, 2H).

## DISCUSSION

We analyzed serum and intratumoral tissues from HCC patients to investigate differential miRNA expression in HCC patients with BM and without BM. We found 90 miRNAs that were differentially expressed in patients with BM compared with non-BM patients. Of the 90 miRNAs, only miR-34a and miR-498 had FDRs<0.05. We further determined that miR-34a levels in serum and intratumoral tissue predicted BM in HCC patients. BM is a common cause of pain and the source of other symptoms that reduce the quality of life for HCC patients [10]. A screening method to predict the risk of BM in HCC patients would allow for improved treatments for HCC patients with a high risk of BM. For example, patients with an increased risk of BM could be treated with bisphosphonates to prevent BM.

Overexpression of miR-34a reportedly suppresses tumor progression and leads to improved prognoses, whereas reduced miR-34a expression is associated with poor overall survival in several cancers [11–17]. The association between miR-34a levels and glioma has been more ambiguous. Gao *et al*. found that lower miR-34a expression was correlated with reduced progression-free survival and overall survival in glioma patients [18], but Genovese *et al*. found that lower miR-34a expression led to improved overall survival in glioblastoma patients [19]. In this study, we found that low levels of miR-34a in serum and intratumoral tissue were associated with increased incidence of BM in HCC patients. We also found that low levels of miR-34a in intratumoral tissue increased BCLC stage and vascular invasion in HCC patients.

Serum miRNAs have been used as noninvasive biomarkers for many cancers [20–23]. MiR-34 has also been found to suppress osteoclasts and has potential as a therapeutic to protect the skeleton and to ameliorate BM [24]. Further, Chen *et al*. found that miR-34a inhibited BM in Ras-activated prostate cancer [25], and Gougelet *et al*. found that miR-34a functioned as an inhibitor and had antitumor activity in liver cancer [26]. Yang *et al*. found a correlation between miR-34a levels and venous metastasis in HBV-positive HCC patients [27]. We found that low miR-34a levels in serum and intratumoral tissue were associated with increased incidence of BM in HCC patients and that miR-34a expression can be used to identify HCC patients at high risk for developing BM.

We believe that miR-34a blocks osteoporosis and BM by inhibiting osteoclastogenesis in HCC patients because miR-34a has been found to directly target transforming growth factor-β-induced factor 2 (TGIF2) and to reduce bone resorption [24]. Because a reduction in miR-34a may be linked to a high level of TGF-β signaling activity [27], we speculate that miR-34a contributes to BM in HCC via a TGF-β-miR-34a signaling pathway. MiR-34a may be used as a biomarker to predict BM in HCC patients; thus, miR-34a could aid in clinical decision-making.

In conclusion, this study demonstrated that low miR-34a expression in serum and in intratumoral tissue are independent risk factors for developing BM in HCC patients. Our findings suggest that miR-34a may be important for facilitating BM in patients with HCC. Nonetheless, further study is required to elucidate the mechanism by which low miR-34a increases the development of BM in HCC patients.

## MATERIALS AND METHODS

### Clinical studies

Ten HCC patients underwent curative resection at Zhongshan Hospital and presented with BM, but had no additional metastases. Blood samples from these patients were collected at the time of surgery. The matched non-BM patients included 10 HCC patients who did not develop metastases 5 years after curative resection. Blood samples from these patients were collected at Zhongshan Hospital between February 2008 and January 2010. To obtain serum samples, whole blood samples were centrifuged at 1000 × *g* for 10 min at 4°C. Serum supernatants were then transferred to new tubes and stored at -80°C.

We used age, gender, hepatitis B surface antigen (HBsAg), hepatitis C antibody (HCV-Ab), α-fetoprotein, alanine transaminase, γ-glutamyl-transferase, liver cirrhosis, Child–Pugh score, tumor differentiation, tumor size, tumor number, tumor encapsulation, vascular invasion, and Barcelona Clinic Liver Cancer (BCLC) stage data to match the BM group with a non-BM group. These factors did not differ significantly between the two groups. Clinicopathologic characteristics are summarized in Supplementary Table S3.

An additional study involving an independent cohort of 106 HCC patients was conducted to evaluate the clinical significance of candidate miRNAs identified in our preliminary serum microarray study. All of the 106 patients underwent a hepatectomy at Zhongshan Hospital. Blood samples were collected from the patients between August 2008 and September 2011 before their hepatectomies. None of the patients had distant metastases at the time of blood collection. To exclude the possibility of extrahepatic spread, each patient underwent a chest X-ray and abdominal ultrasonography before surgery, and a bone scan was performed if the patient reported bone pain. If extrahepatic spread was suspected, computed tomography and/or magnetic resonance imaging (MRI) were used to determine whether extrahepatic spread had occurred. Additional inclusion criteria were as follows: HCC diagnosis based on pathology, no prior anticancer treatment, acceptable blood sample, and complete clinicopathologic follow-up data. The tumor stage was determined according to the BCLC staging system. The histologic grade of tumor differentiation was assigned using the Edmondson grading system. Tumor size was determined by measuring the largest dimension of the tumor specimen. The extent of vascular invasion was determined by microscopic examination of the resected specimen. The detailed clinicopathologic features of these patients are provided in Supplementary Table S4.

The clinical significance of the candidate biomarker was evaluated using intratumoral tissues from an independent cohort of 296 consecutive HCC patients who did not have distant metastasis before surgery. All of these patients underwent a hepatectomy between August 2002 and September 2007 at the Liver Cancer Institute, Zhongshan Hospital, Fudan University. None of these patients received any preoperative anticancer treatment, and prior to surgery, each patient underwent a chest X-ray and abdominal ultrasonography to exclude extrahepatic metastases. The clinicopathological characteristics of these patients are summarized in Supplementary Table S5.

Study protocols were approved by the Zhongshan Hospital Research Ethics Committee. Informed consent was obtained from each patient in accordance with the committee regulations.

### Follow-up and postoperative treatment

Serum samples were collected from 10 HCC patients that presented with BM but had no additional metastases. The matched non-BM group included 10 HCC patients who also had no additional metastases at follow-up; sera from the non-BM patients were collected at follow-up. The median follow-up time was 65 months with a range of 60–73 months.

The independent cohort of 106 HCC patients received their follow-ups by August 2015, and the median follow-up time was 41.9 months with a range of 4.4–84 months.

The clinical significance of the candidate miRNA was evaluated using intratumoral tissues from an independent cohort of 296 consecutive HCC patients. These patients were observed until December 2012. The median follow-up time for this group was 54.6 months with a range of 3.2–124.1 months. At each check-up, a medical history was recorded and a physical examination was performed. A chest radiograph was performed every 6 months, ultrasonography of the abdomen was performed every 3 months, and laboratory tests were performed every 3 months. Time to recurrence was calculated from the date of the operation to the date of recurrence. BM was diagnosed as previously described [4]. Briefly, a bone scan was performed annually. If a patient reported localized bone pain, a bone scan or an MRI was performed immediately. A BM diagnosis was based on a history of HCC, the presence of symptoms, and radiological imaging studies. Time to BM was calculated from the date of the operation to the date of BM. When a diagnosis of BM was made, the affected bone was treated with external beam radiotherapy. Metastases at other locations were treated with radiotherapy, interventional therapy, or surgery.

### MiRNA microarray analysis

Serum samples from 20 patients (10 BM and 10 non-BM) were used for miRNA profiling. Profiling was performed using the 7th generation miRCURY^TM^ LNA Array (v.18.0, Exiqon, Vedbaek, Denmark) and microarray experiments were performed by KangChen Bio-tech (Shanghai, China). Briefly, total RNA was harvested using TRIzol^®^ LS reagent (Invitrogen) and a miRNeasy mini kit (Qiagen) according to the manufacturer's instructions. In detail, 0.75 ml TRIzol^®^ LS reagent was added to 0.25 ml serum samples. Chloroform (0.2 ml) was added for phase separation, 1.5 volumes of 100% ethanol were added to the aqueous phase, and the mixture was loaded onto a miRNeasy column according to the manufacturer's instructions. RNA samples were quantified using a NanoDrop^®^ 1000 (ND-1000, NanoDrop Technologies), labeled using the miRCURY LNA™ Hy3™/Hy5™ Power labeling kit, and hybridized on the miRCURY LNA™ Array (v.18.0). After washing, the slides were scanned using the Axon GenePix^®^ 4000B microarray scanner. Scanned images were imported into GenePix Pro 6.0 software (Axon) for grid alignment and data extraction. Replicated miRNAs were averaged and miRNAs with intensities ≥ 30 in all samples were used to calculate the normalization factor according to the median normalization method. After normalization, significant differentially expressed miRNAs were identified by Volcano Plot filtering. Finally, hierarchical clustering was performed using MEV software (v.4.6, TIGR) to identify differences in miRNA expression profiles among samples.

### RNA isolation and qRT-PCR

Total RNA was isolated using TRIzol^®^ LS reagent (Invitrogen) and a miRNeasy mini kit (Qiagen) according to manufacturer's instructions. Briefly, 3 volumes (0.75 ml) of TRIzol^®^ LS reagent was added to a serum sample (0.25 ml), 25 fmol of cel-miR-39 was added for normalization [28], 0.2 ml of chloroform was added, and each sample was centrifuged. After centrifugation, the aqueous phase was transferred to a new tube, 1.5 volumes of 100% ethanol were added, and the mixture was loaded onto a miRNeasy column (Qiagen) according to the manufacturer's instructions. The final elution volume ranged from 20–30 μl. RNA sample concentrations were quantified using a NanoVue Plus spectrophotometer (GE, General Electric Company). Serum RNA (500 ng, including miRNA) was used for first-strand DNA synthesis with an All-in-One™ First-Strand cDNA Synthesis kit (GeneCopoeia Inc.). Real-time PCR was performed in triplicate using the SYBR^®^ Green PCR method and the All-in-One™ miRNA qPCR Detection kit (GeneCopoeia Inc.). The expression levels of serum miRNAs were normalized against cel-miR-39 using the 2^−ΔΔCt^ method [29]. Total RNA was isolated from HCC cell lines using TRIzol^®^ (Invitrogen) and miR-34a expression was normalized against U6 expression. Expression analysis was performed using a 7500 Real-Time PCR System (Applied Biosystems).

### Tissue microarray

A tissue microarray (TMA) was constructed as previously described [30–32]. Hematoxylin and eosin (H&E)-stained slides were screened to identify the optimal intratumoral tissue for analysis. The TMA slides were then constructed (in collaboration with the Shanghai Biochip Company Ltd.) using samples from the cohort of 296 HCC patients. From each FFPE HCC tissue sample, two punch cores (longest dimension = 1.0 mm) were collected from the non-necrotic areas of the tumor foci. TMA sections (4 mm) were constructed using the intratumoral samples from the 296 HCC patients.

### *In situ* hybridization

*In situ* hybridization (ISH) was performed using miR-34a 5’-DIG-labeled LNA™ probes (Exiqon, Vedbaek, Denmark) according to the manufacturer's protocol. The sequence of the detection probe for human mature miR-34a was 5’ ACAACCAGCTAAGACACTGCCA 3’. TMAs composed of intratumoral tissues from 296 patients were prepared as follows: the TMAs were (1) acetylated with 0.25% acetic anhydride after the sections were dewaxed and rehydrated; (2) prehybridized in Exiqon hybridization buffer (Exiqon, Vedbæk, Denmark) at 55°C for 60 min; (3) incubated with 40 μl of a 1:600 dilution of the corresponding 5’-DIG-labeled LNA™ miRNA probe; (4) washed twice with SSC buffer at 60°C for a minimum of 20 min; (5) treated with digoxigenin (DIG) blocking reagent (Invitrogen); (6) incubated with anti-DIG alkaline phosphatase conjugated antibody (Invitrogen) at 4°C for 24 h; and (7) washed twice and counterstained slightly with Mayer's hematoxylin.

### Evaluation of ISH findings

The slides were examined by three independent, blinded observers using an Olympus BX51 microscope (Olympus BX51, CCD: DP71, Japan). The intensity of the staining and the percentage of positively stained tumor cells were scored by each observer, and the three scores were averaged [33]. The scoring scale for the percentage of positively stained cells was as follows: none = 0; <1% = 1; 1–10% = 2; 10–33% = 3; 34–67% = 4; and > 67% = 5. The intensity of the staining was scored semiquantitatively as follows: none = 0; weak = 1; intermediate = 2; and strong = 3. The staining intensity and the percent of positively stained cells scores were summed to yield a final score that ranged from 0–8. A final score of 0–2 was designated as negative for miR-34a expression and a final score of 3–8 was designated as positive for miR-34a expression.

### Cell culture

The SMMC-7721, HCCLM3, and MHCC97H human hepatoma cell lines used in this study were purchased from KeyGen (Nanjing KeyGen Biotech Co.Ltd, China), and the HepG2 human hepatoma cells were purchased from the Chinese Academy of Sciences cell bank. The HCCLM3 cells were previously described [34]. All of the cell lines were maintained in DMEM medium (GIBCO, Carlsbad, CA, USA) supplemented with 10% fetal bovine serum, 80 U/ml of penicillin sodium, and 0.08 mg/ml of streptomycin sulfate at 37°C in humidified air containing 5% carbon dioxide.

### Oligonucleotide transfection

The miRIDIAN miR-34a Hairpin Inhibitor, Mimic, and negative control oligonucleotides (Dharmacon, GE Healthcare) were used to inhibit and restore miR-34a function. These oligonucleotides were transfected into HCC cells using Lipofectamine™ 2000 according to the manufacturer's instructions (Invitrogen).

### *In vitro* cell proliferation assays

Cell proliferation was detected using the Cell Counting Kit-8 (KeyGen Biotech Co.Ltd, China) assay. In brief, SMMC-7721 and HCCLM3 cells were seeded into 96-well plates at an initial density of 1×10^4^ cells per well. The oligonucleotides were transfected into the HCC cells 24 h later, and at 0, 24, 48, 72, and 96 h after oligonucleotide transfection, 10 μl of the kit reagent was added to each well. All of the plates were scanned at 450 nm 2 h later using a microplate reader (Molecular Devices, USA) and the cell proliferation value was calculated based on the resulting OD value.

### *In vitro* migration and invasion assays

Cell migration and invasion were analyzed using 24-well transwell plates (8-μm pore size, Corning, USA). For the transwell migration assays, 5 × 10^4^ cells were seeded in the upper chamber of the plate with a non-coated membrane. For the invasion assays, 1.0 × 10^5^ cells were seeded in the upper chamber of the plate with a Matrigel^®^-coated membrane (Corning, USA). The cells were seeded in 1% serum medium, and 10% serum medium was used as the chemoattractant in the lower chamber of the plate. After incubation at 37°C for 24 or 48 h, nonmigratory and noninvasive cells in the upper chambers were removed using cotton wool. The invading cells on the undersides of the membranes were fixed in 100% methanol for 10 min, stained with Giemsa (Jiancheng, Nanjing, China), and counted by microscopy. Sixty hours prior to plating, miR-34a inhibitors, mimics, or negative controls were transfected into cells at a final concentration of 100 nM using Lipofectamine™ 2000.

### Statistical analysis

All of the statistical analyses were performed using IBM SPSS Statistics 20. The Fisher's exact test was used to compare qualitative variables, and quantitative variables were analyzed using Pearson's correlation test. To determine whether there was an association between the expression of the candidate molecule and a higher incidence of BM, we analyzed censored time-to-event data using a log-rank test and a Cox regression model. A two-tailed *P*< 0.05 was considered significant. A correction for multiple hypotheses testing was performed using the false discovery rate (FDR).

## SUPPLEMENTARY FIGURES AND TABLES




