# Correction: MicroRNA-34a expression levels in serum and intratumoral tissue can predict bone metastasis in patients with hepatocellular carcinoma

**DOI:** 10.18632/oncotarget.27296

**Published:** 2019-10-29

**Authors:** Zuo-Lin Xiang, Xiao-Mei Zhao, Li Zhang, Ping Yang, Jia Fan, Zhao-You Tang, Zhao-Chong Zeng

**Affiliations:** ^1^ Department of Radiation Oncology, Zhongshan Hospital, Fudan University, Shanghai, China; ^2^ Department of Liver Cancer Institute, Zhongshan Hospital, Fudan University, Shanghai, China

**This article has been corrected:** Due to errors in image processing, there were misplaced images in Figure 2. An updated Figure 2 using the original data is shown below. The authors declare that these corrections do not change the results or conclusions of this paper.

Original article: Oncotarget. 2016; 7:87246–87256. 87246-87256. https://doi.org/10.18632/oncotarget.13531

**Figure 2:**
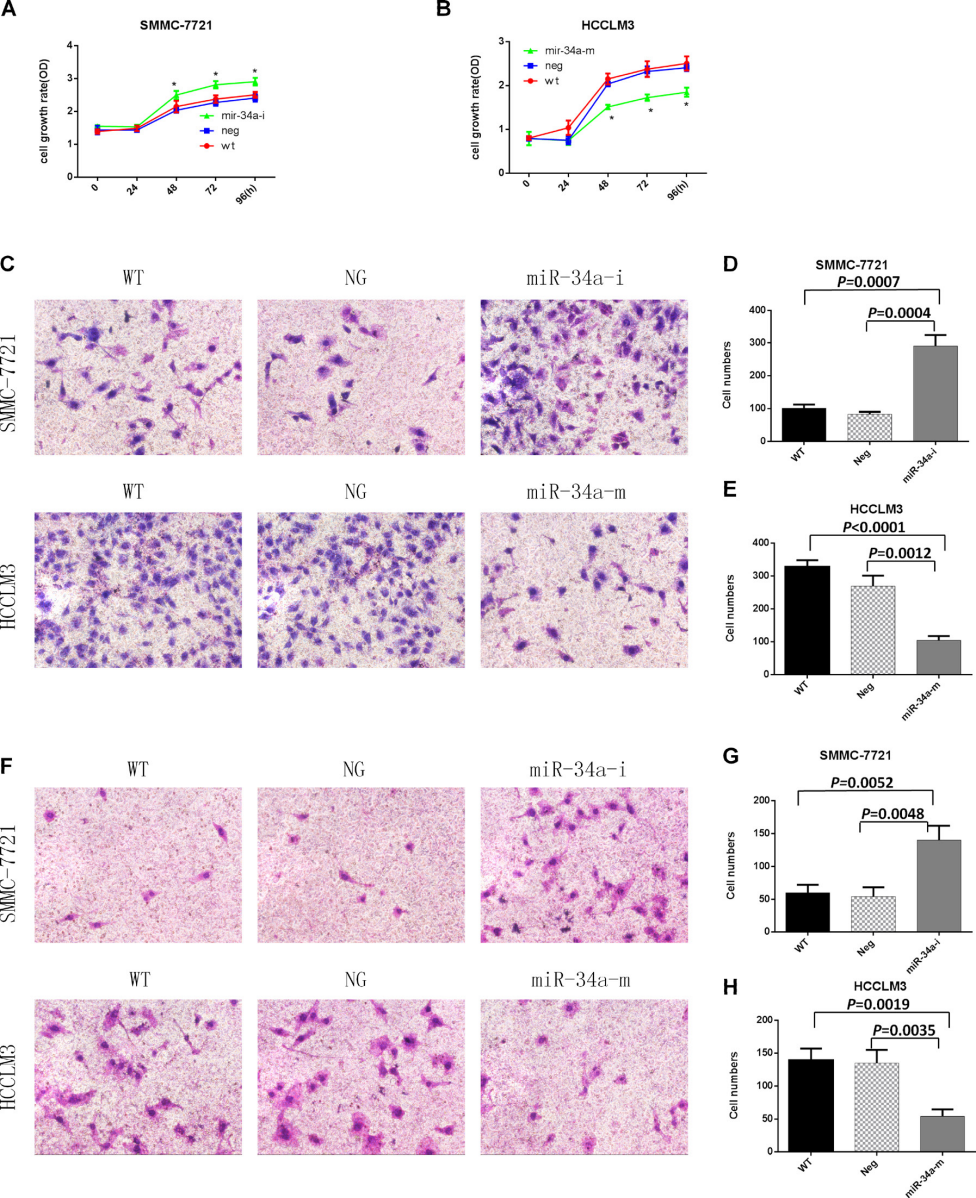
Proliferation, migration, and invasion assays of SMMC-7721 and HCCLM3 cells after transfection with the indicated oligonucleotides. WT, NG, miR-34a-m, and miR-34a-i indicate non-transfected, negative control oligonucleotide-transfected, miR-34a mimic- transfected, and miR-34a inhibitor-transfected cells, respectively. (**A**) SMMC-7721 cell growth rates after transfection with the miR-34a inhibitor. (**B**) HCCLM3 cell growth rates after transfection with the miR-34a mimic. * = *P*<0.05, compared to negative controls at the same time point. (**C**) Representative images of the migration assay. (**D**) and (**E**) Numbers of migrating cells on the undersides of the membranes. Data are presented as mean ± SEM and are representative of three independent experiments. The images were acquired at a magnification of 200×. (**F**) Representative images of the invasion assay. (**G**) and (**H**) Numbers of invading cells on the undersides of the membranes. Data are presented as mean ± SEM and are representative of three independent experiments. The images were acquired at a magnification of 200×.

