# Supplementary material for: MicroRNA-34a expression levels in serum and intratumoral tissue can predict bone metastasis in patients with hepatocellular carcinoma
Source: Oncotarget. 2016 Nov 23;7(52):87246–56. doi: 10.18632/oncotarget.13531 (PMC5349985; doi:10.18632/oncotarget.13531)
Supplement: Supplementary file 1 [file oncotarget-07-87246-s001.pdf]

MicroRNA-34a expression levels in serum and intratumoral tissue can predict bone metastasis in patients with hepatocellular carcinoma

SUPPLEMENTARY FIGURES AND TABLES

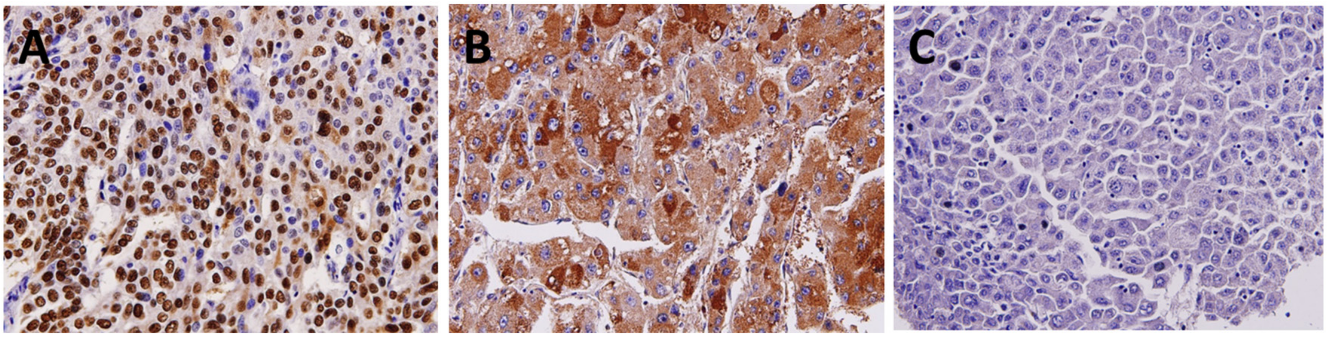

Supplementary Figure S1: MicroRNA-34a expression in TMAs from HCC patients. A. miRNA-34a was expressed at high levels in the nuclei of some tumor cells. **B.** miRNA-34a was expressed at high levels in the cytoplasm of some tumor cells. **C.** miRNA-34a was expressed at low levels in some tumor cells. The images were acquired at a magnification of 200×.

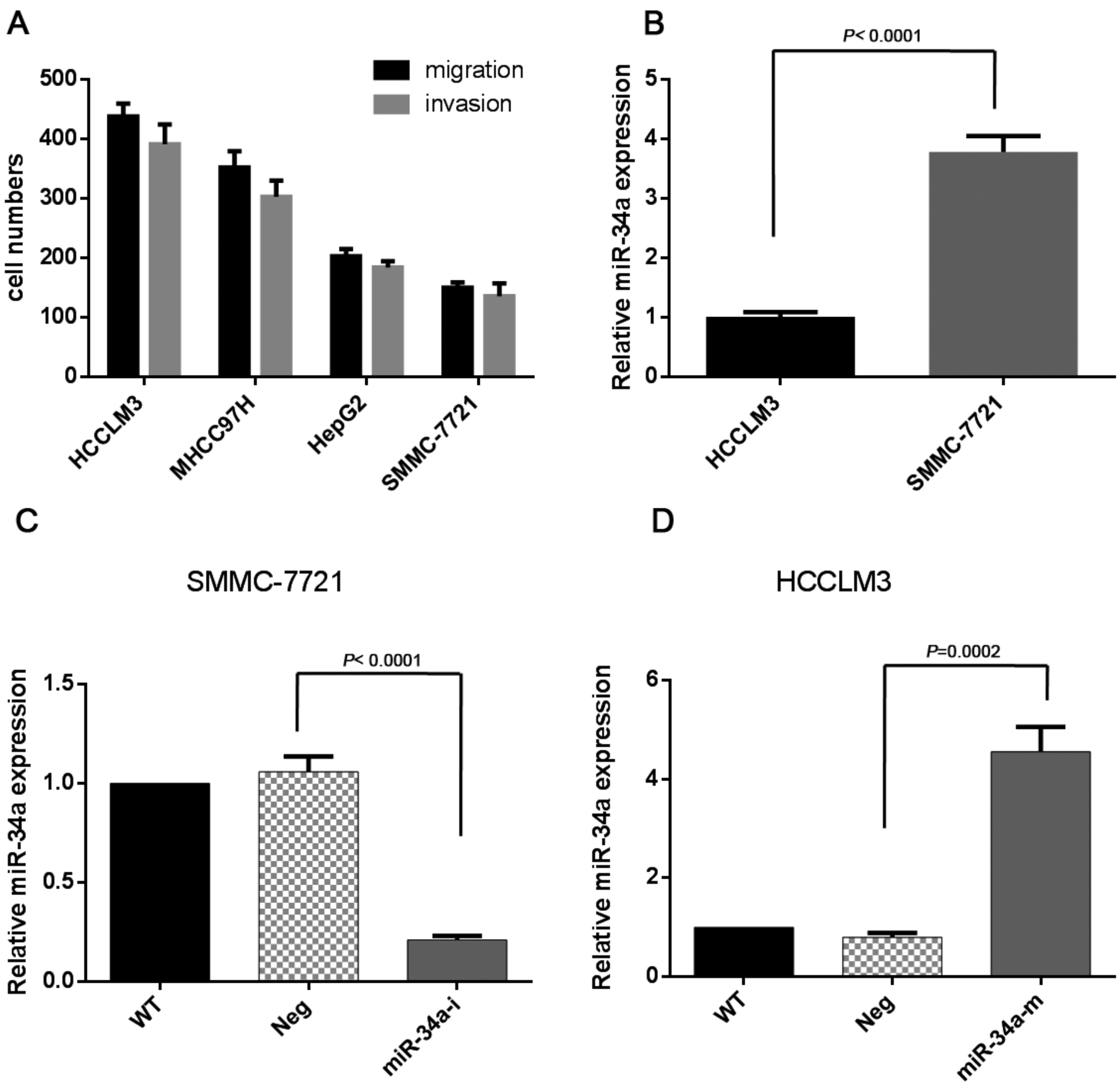

Supplementary Figure S2: MiR-34a expression in SMMC-7721 and HCCLM3 cells. WT, NG, miR-34a-m, and miR-34a-i indicate non-transfected, negative control oligonucleotide- transfected, miR-34a mimic-transfected, and miR-34a inhibitor-transfected cells, respectively. **A.** Transwell analysis to detect migration and invasion capabilities of HCC cells. **B.** Basal miR-34a expression in SMMC-7721 and HCCLM3 cells. **C.** MiR-34a expression in SMMC-7721 cells after transient transfection with the indicated oligonucleotides. **D.** MiR-34a expression in HCCLM3 cells after transient transfection with the indicated oligonucleotides. Data are presented as mean ± SEM and are representative of three independent experiments.

Supplementary Table S1: The list of 90 differentially expressed miRNAs (change 1.5 fold as a cut-off level) in serum samples from 10 BM HCC patients compared to 10 NBM HCC patients

See Supplementary File 1

Supplementary Table S2: Correlation between microRNA-34a expression and the clinicopathologic features of 296 patients

Clinicopathologic Parameters	N	microRNA-34a Levels (%)		
		negative	positive	*P*
Age				
≤ 51	152	41 (53.2)	111 (50.7)	0.791
>51	144	36 (46.8)	108 (49.3)	
Gender				
male	258	68 (88.3)	190 (86.8)	0.844
female	38	9 (11.7)	29 (13.2)	
HBsAg				
negative	68	23 (29.9)	45 (20.5)	0.115
positive	228	54 (70.1)	174 (79.5)	
HCV-Ab				
negative	291	75 (97.4)	216 (98.6)	0.608
positive	5	2 (2.6)	3 (1.4)	
AFP				
≤ 20	87	21 (27.3)	66 (30.1)	0.666
>20	209	56 (72.7)	153 (69.9)	
ALT				
≤ 40	177	39 (50.6)	138 (63.0)	0.060
>40	119	38 (49.4)	81 (37.0)	
γ-GT				
≤ 50	107	25 (32.5)	82 (37.4)	0.491
>50	189	52 (67.5)	137 (62.6)	
Liver cirrhosis				
no	42	7 (9.1)	35 (16.0)	0.183
yes	254	70 (90.9)	184 (84.0)	
Child-Pugh score				
A	294	76 (98.7)	218 (99.5)	0.453
B	2	1 (1.3)	1 (0.5)	
Tumor differentiation				
I–II	210	50 (64.9)	160 (73.1)	0.191
III–IV	86	27 (35.1)	59 (26.9)	
Tumor size, cm				
≤ 5	149	40 (51.9)	109 (49.8)	0.792
>5	147	37 (48.1)	110 (50.2)	
Tumor number				
single	210	49 (63.6)	161 (73.5)	0.110
multiple	86	28 (36.4)	58 (26.5)	
Tumor encapsulation				
complete	155	46 (59.7)	109 (49.8)	0.146
none	141	31 (40.3)	110 (50.2)	
Vascular invasion				
no	239	56 (72.7)	183 (83.6)	0.044
yes	57	21 (27.3)	36 (16.4)	
BCLC stage				
0-A	248	57 (74.0)	191 (87.2)	0.011
B-C	48	20 (26.0)	28 (12.8)	

Tumor differentiation evaluation was based on Edmondson-Steiner grading.
Abbreviations: HBsAg, hepatitis B virus surface antigen; HCV-Ab, hepatitis C virus antibody; AFP, alpha-fetoprotein; ALT, alanine aminotransferase; γ-GT, γ-glutamyl-transferase; BCLC, Barcelona Clinic Liver Cancer.

Supplementary Table S3: The clinicopathologic characteristics of 10 HCC with BM and matched 10 HCC NBM who went along miRNA microarray

variable	No. of patients(%)		*P*
	BM (N=10) (%)	NBM (N=10)(%)	
age			
≤51	5 (50)	7 (70)	0.650
>51	5 (50)	3 (30)	
gender			
male	8 (80)	9 (90)	1.000
female	2 (20)	1 (10)	
HBsAg			
negtive	1 (10)	3 (30)	0.582
positive	9 (90)	7 (70)	
HCV-Ab			
negtive	10 (100)	10 (100)	1.000
positive	0 (0)	0 (0)	
AFP(ng/ml)			
≤20	4 (40)	3 (30)	1.000
>20	6 (60)	7 (70)	
ALT(U/L)			
≤40	4 (40)	3 (30)	1.000
>40	6 (60)	7 (70)	
γ-GT			
≤ 50	3 (30)	5 (50)	0.650
>50	7 (70)	5 (50)	
Liver cirrhosis			
no	2 (20)	1 (10)	1.000
yes	8 (80)	9 (90)	
Child-Pugh score			
A	10 (100)	10 (100)	NA
B	0 (0)	0 (0)	
Tumor differentiation			
≤ 5	6 (60)	7 (70)	1.000
>5	4 (40)	3 (30)	
Tumor size(cm)			
≤5	6 (60)	4 (40)	0.656
>5	4 (40)	6 (60)	
Tumor number			
single	5 (50)	6 (60)	1.000
multiple	5 (50)	4 (40)	
Tumor encapsulation			
complete	4 (40)	6 (60)	0.656
none	6 (60)	4 (40)	
Vascular invasion			
no	6 (60)	8 (80)	0.628
yes	4 (40)	2 (20)	
BCLC stage			
0-A	8 (80)	9 (90)	1.000
B-C	2 (20)	1 (10)	

Tumor differentiation evaluation was based on Edmondson-Steiner grading.
Abbreviations: HBsAg, hepatitis B virus surface antigen; HCV-Ab, hepatitis C virus antibody; AFP, alpha-fetoprotein; ALT, alanine aminotransferase; γ-GT, γ-glutamyl-transferase; BCLC, Barcelona Clinic Liver Cancer

P value: Fisher exact test (two tailed).

Supplementary Table S4: Clinicopathological characteristics of 106 hepatocellular carcinoma patients

Variable	*n* of patients (%)
Age	
≤ 51	51 (48.1)
>51	55 (51.9)
Gender	
male	91 (85.8)
female	15 (14.2)
HBsAg	
negative	22 (20.8)
positive	84 (79.2)
HCV-Ab	
negative	104 (98.1)
positive	2 (1.9)
AFP	
≤ 20	28 (26.4)
>20	78 (73.6)
ALT	
≤ 40	65 (61.3)
>40	41 (38.7)
γ-GT	
≤ 50	41 (38.7)
>50	65 (61.3)
Liver cirrhosis	
no	14 (13.2)
yes	92 (86.8)
Child-Pugh score	
A	105 (99.0)
B	1 (1.0)
Tumor differentiation	
I–II	73 (68.9)
III–IV	33 (31.1)
Tumor size, cm	
≤ 5	55 (51.9)
>5	51 (48.1)
Tumor number	
single	75 (70.8)
multiple	31 (29.2)
Tumor encapsulation	
complete	54 (50.9)
none	52 (49.1)
Vascular invasion	
no	82 (77.4)
yes	24 (22.6)
BCLC stage	
0-A	86 (71.1)
B-C	20 (18.9)

Tumor differentiation evaluation was based on Edmondson-Steiner grading.
Abbreviations: HBsAg, hepatitis B virus surface antigen; HCV-Ab, hepatitis C virus antibody; AFP, alpha-fetoprotein; ALT, alanine aminotransferase; γ-GT, γ-glutamyl-transferase; BCLC, Barcelona Clinic Liver Cancer.

Supplementary Table S5: Clinicopathological characteristics of 296 hepatocellular carcinoma patients

Variable	*n* of patients (%)
Age	
≤ 51	152 (51.4)
>51	144 (48.6)
Gender	
male	258 (87.2)
female	38 (12.8)
HBsAg	
negative	68 (23.0)
positive	228 (77.0)
HCV-Ab	
negative	291 (98.3)
positive	5 (1.7)
AFP	
≤ 20	87 (29.4)
>20	209 (70.6)
ALT	
≤ 40	177 (59.8)
>40	119 (40.2)
γ-GT	
≤ 50	107 (36.1)
>50	189 (63.9)
Liver cirrhosis	
no	42 (14.2)
yes	254 (85.8)
Child-Pugh score	
A	294 (99.3)
B	2 (0.7)
Tumor differentiation	
I–II	210 (70.9)
III–IV	86 (29.1)
Tumor size, cm	
≤ 5	149 (50.3)
>5	147 (49.7)
Tumor number	
single	210 (70.9)
multiple	86 (29.1)
Tumor encapsulation	
complete	155 (52.4)
none	141 (47.6)
Vascular invasion	
no	239 (80.7)
yes	57 (19.3)
BCLC stage	
0-A	248 (83.8)
B-C	48 (16.2)

Tumor differentiation evaluation was based on Edmondson-Steiner grading.
Abbreviations: HBsAg, hepatitis B virus surface antigen; HCV-Ab, hepatitis C virus antibody; AFP, alpha-fetoprotein; ALT, alanine aminotransferase; γ-GT, γ-glutamyl-transferase; BCLC, Barcelona Clinic Liver Cancer.
