# Supplementary material for: MicroRNA-34a expression levels in serum and intratumoral tissue can predict bone metastasis in patients with hepatocellular carcinoma
Source: Oncotarget. 2016 Nov 23;7(52):87246–56. doi: 10.18632/oncotarget.13531 (PMC5349985; doi:10.18632/oncotarget.13531)
Supplement: Supplementary file 2 [file oncotarget-07-87246-s002.docx]

Supplementary Table 1: The list of 90 differentially expressed miRNAs (change 1.5 fold as a cut-off level) in serum samples from 10 BM HCC patients compared to 10 NBM HCC patients

| **microRNA** | **MirBase no** | | **Fold change** | | **P-value** | | **FDR** |
| --- | --- | --- | --- | --- | --- | --- | --- |
| upregulated 27 miRNAs | |  | |  | |  | |
| hsv1-miR-H6-3p | MIMAT0008404 | | 1.97 | | 0.00486 | | 0.142 |
| hsa-miR-125b-1-3p | MIMAT0004592 | | 2.53 | | 0.01214 | | 0.221 |
| hsa-miR-4472 | MIMAT0018999 | | 1.88 | | 0.00231 | | 0.113 |
| hsv2-miR-H9-3p | MIMAT0014352 | | 1.98 | | 0.04003 | | 0.318 |
| hsa-miR-4290 | MIMAT0016921 | | 2.11 | | 0.00206 | | 0.113 |
| hsa-miR-642b-5p | MIMAT0022736 | | 2.21 | | 0.01654 | | 0.244 |
| hsa-miR-4755-5p | MIMAT0019895 | | 2.88 | | 0.02968 | | 0.294 |
| hsa-miR-4508 | MIMAT0019045 | | 2.24 | | 0.00221 | | 0.113 |
| hsa-miR-548at-5p | MIMAT0022277 | | 2.43 | | 0.00827 | | 0.191 |
| hsa-miR-4505 | MIMAT0019041 | | 6.63 | | 0.00674 | | 0.175 |
| hsa-miR-4685-3p | MIMAT0019772 | | 3.18 | | 0.00116 | | 0.088 |
| hsa-miR-1273a | MIMAT0005926 | | 4.38 | | 0.00452 | | 0.142 |
| hsa-miR-3162-3p | MIMAT0019213 | | 3.14 | | 0.00037 | | 0.068 |
| hsa-miR-498 | MIMAT0002824 | | 1.73 | | 1.07E-05 | | 0.018 |
| hsa-miR-3940-5p | MIMAT0019229 | | 1.68 | | 0.0095 | | 0.205 |
| hsa-miR-1827 | MIMAT0006767 | | 1.6 | | 0.01449 | | 0.227 |
| hsa-miR-660-3p | MIMAT0022711 | | 1.63 | | 0.04939 | | 0.354 |
| hsa-miR-5095 | MIMAT0020600 | | 5.97 | | 0.01225 | | 0.221 |
| hsa-miR-4258 | MIMAT0016879 | | 1.57 | | 0.04513 | | 0.338 |
| hsa-miR-3189-3p | MIMAT0015071 | | 3.6 | | 0.00227 | | 0.113 |
| hsa-miR-1587 | MIMAT0019077 | | 5.14 | | 0.03975 | | 0.318 |
| hsa-miR-4455 | MIMAT0018977 | | 1.96 | | 0.00393 | | 0.133 |
| hsa-miR-4707-5p | MIMAT0019807 | | 1.77 | | 0.01045 | | 0.212 |
| hsa-miR-1246 | MIMAT0005898 | | 4.64 | | 0.02174 | | 0.278 |
| hsa-miR-1273g-3p | MIMAT0022742 | | 3.54 | | 0.02759 | | 0.287 |
| hsa-miR-4454 | MIMAT0018976 | | 3.29 | | 0.00309 | | 0.123 |
| hsa-miR-4632-3p | MIMAT0019688 | | 1.78 | | 0.01361 | | 0.221 |
| downregulated 63 miRNAs | |  | |  | |  | |
| hsa-miR-508-5p | [MIMAT0004778](http://www.mirbase.org/cgi-bin/mature.pl?mature_acc=MIMAT0004778) | | 0.12 | | 0.00213 | | 0.113 |
| hsa-miR-150-5p | MIMAT0000451 | | 0.58 | | 0.0252 | | 0.281 |
| hsa-miR-4748 | MIMAT0019884 | | 0.32 | | 0.00274 | | 0.117 |
| hsa-miR-4726-5p | MIMAT0019845 | | 0.65 | | 0.00133 | | 0.092 |
| kshv-miR-K12-2-5p | MIMAT0002183 | | 0.17 | | 0.00595 | | 0.162 |
| kshv-miR-K12-12-3p | [MIMAT0015238](http://www.mirbase.org/cgi-bin/mature.pl?mature_acc=MIMAT0015238) | | 0.36 | | 0.02639 | | 0.286 |
| hsa-miR-3915 | MIMAT0018189 | | 0.44 | | 0.01352 | | 0.221 |
| hsa-miR-423-5p | MIMAT0004748 | | 0.59 | | 0.03406 | | 0.314 |
| ebv-miR-BART19-5p | MIMAT0004836 | | 0.54 | | 0.04536 | | 0.338 |
| hsa-miR-26b-3p | MIMAT0004500 | | 0.36 | | 0.02823 | | 0.292 |
| hsa-miR-450b-3p | MIMAT0004910 | | 0.3 | | 0.0349 | | 0.314 |
| hsa-miR-4317 | MIMAT0016872 | | 0.25 | | 0.00839 | | 0.191 |
| hsa-miR-532-3p | MIMAT0004780 | | 0.54 | | 0.03015 | | 0.295 |
| hsa-miR-296-5p | MIMAT0000690 | | 0.46 | | 0.01479 | | 0.23 |
| hsa-miR-4783-3p | MIMAT0019947 | | 0.26 | | 0.00705 | | 0.178 |
| hsa-miR-652-5p | MIMAT0022709 | | 0.41 | | 0.00221 | | 0.113 |
| hsa-miR-605-5p | MIMAT0003273 | | 0.12 | | 0.00105 | | 0.088 |
| hsa-miR-431-5p | MIMAT0001625 | | 0.39 | | 0.03947 | | 0.318 |
| hsa-miR-4450 | MIMAT0018971 | | 0.2 | | 0.02449 | | 0.281 |
| hsa-miR-4695-3p | MIMAT0019789 | | 0.23 | | 0.03453 | | 0.314 |
| hsa-miR-513b-5p | MIMAT0005788 | | 0.29 | | 0.00245 | | 0.113 |
| hsa-miR-3611 | MIMAT0017988 | | 0.64 | | 0.02521 | | 0.281 |
| hsa-miR-4712-3p | MIMAT0019819 | | 0.52 | | 0.01812 | | 0.255 |
| hsa-miR-4513 | MIMAT0019050 | | 0.45 | | 0.03227 | | 0.308 |
| hsa-miR-371b-3p | MIMAT0019893 | | 0.51 | | 0.00152 | | 0.098 |
| hsa-miR-125a-5p | MIMAT0000443 | | 0.27 | | 0.0038 | | 0.132 |
| hsa-miR-513a-5p | MIMAT0002877 | | 0.57 | | 0.01343 | | 0.221 |
| hsa-miR-642b-3p | MIMAT0018444 | | 0.43 | | 0.046 | | 0.34 |
| hsa-miR-1249 | MIMAT0005901 | | 0.65 | | 0.04011 | | 0.318 |
| hsa-miR-433-5p | MIMAT0026554 | | 0.23 | | 0.00154 | | 0.098 |
| hsa-miR-3150a-3p | MIMAT0015023 | | 0.13 | | 0.03874 | | 0.318 |
| hsa-miR-1185-2-3p | MIMAT0022713 | | 0.32 | | 0.02272 | | 0.278 |
| hsa-miR-711 | MIMAT0012734 | | 0.42 | | 0.00891 | | 0.198 |
| hsa-miR-4669 | MIMAT0019749 | | 0.21 | | 0.02266 | | 0.278 |
| hsa-miR-4506 | MIMAT0019042 | | 0.34 | | 0.0426 | | 0.331 |
| hsa-miR-34a | [MIMAT0000255](http://www.mirbase.org/cgi-bin/mature.pl?mature_acc=MIMAT0000255) | | 0.17 | | 3.58772E-05 | | 0.02 |
| hsa-miR-4646-3p | MIMAT0019708 | | 0.65 | | 0.02451 | | 0.281 |
| hsa-miR-675-3p | MIMAT0006790 | | 0.36 | | 0.00023 | | 0.055 |
| hsa-miR-1825 | MIMAT0006765 | | 0.61 | | 0.03744 | | 0.318 |
| hsa-miR-635 | MIMAT0003305 | | 0.39 | | 0.00269 | | 0.117 |
| hsv1-miR-H6-5p | MIMAT0015281 | | 0.54 | | 0.00797 | | 0.189 |
| hsa-miR-3685 | MIMAT0018113 | | 0.5 | | 0.00697 | | 0.178 |
| hsa-miR-183-3p | MIMAT0004560 | | 0.15 | | 0.01528 | | 0.235 |
| hiv1-miR-H1 | MIMAT0004480 | | 0.47 | | 0.01379 | | 0.221 |
| hsa-miR-1471 | MIMAT0007349 | | 0.27 | | 0.01968 | | 0.266 |
| hsa-miR-4758-5p | MIMAT0019903 | | 0.21 | | 0.00058 | | 0.088 |
| hsa-miR-371a-5p | MIMAT0004687 | | 0.29 | | 0.03268 | | 0.311 |
| hsa-miR-505-5p | MIMAT0004776 | | 0.31 | | 0.00069 | | 0.088 |
| hsa-miR-99b-3p | MIMAT0004678 | | 0.37 | | 0.01348 | | 0.221 |
| hsa-miR-629-5p | MIMAT0004810 | | 0.46 | | 0.02735 | | 0.287 |
| hsa-miR-3191-5p | MIMAT0022732 | | 0.22 | | 0.01121 | | 0.215 |
| hsa-miR-3158-5p | MIMAT0019211 | | 0.31 | | 0.04153 | | 0.324 |
| hsa-miR-7-5p | MIMAT0000252 | | 0.4 | | 0.0251 | | 0.281 |
| hsa-miR-1281 | MIMAT0005939 | | 0.54 | | 0.00473 | | 0.142 |
| hsa-miR-373-5p | MIMAT0000725 | | 0.39 | | 0.00111 | | 0.088 |
| hsa-miR-328-3p | MIMAT0000752 | | 0.55 | | 0.02947 | | 0.294 |
| hsa-miR-378a-3p | MIMAT0000732 | | 0.21 | | 0.02458 | | 0.281 |
| hsa-miR-194-3p | MIMAT0004671 | | 0.48 | | 0.04987 | | 0.354 |
| hsa-miR-451b | MIMAT0019840 | | 0.11 | | 0.0459 | | 0.34 |
| hsa-miR-548k | MIMAT0005882 | | 0.45 | | 0.04378 | | 0.336 |
| hsv2-miR-H6-5p | MIMAT0015651 | | 0.56 | | 0.02233 | | 0.278 |
| hsa-miR-328-5p | MIMAT0026486 | | 0.36 | | 0.0492 | | 0.354 |
| hsa-miR-4739 | MIMAT0019868 | | 0.53 | | 0.03807 | | 0.318 |
